# Robotic-assisted mitral valve surgery without aortic cross-clamping: a safe and feasible technique

**DOI:** 10.3389/fcvm.2023.1111496

**Published:** 2023-05-30

**Authors:** Eyüp Murat Ökten, Zeynep Sıla Özcan, Gökhan Arslanhan, Şahin Şenay, Ahmet Ümit Güllü, Muharrem Koçyiğit, Aleks Değirmencioğlu, Cem Alhan

**Affiliations:** ^1^ Department of Cardiovascular Surgery, Acibadem Mehmet Ali Aydınlar University School of Medicine, Istanbul, Türkiye; ^2^ Department of Cardiovascular Surgery, Acıbadem Maslak Hospital, Istanbul, Türkiye; ^3^ Department of Anesthesiology, Acibadem Mehmet Ali Aydınlar University School of Medicine, Istanbul, Türkiye; ^4^ Department of Cardiology, Acibadem Mehmet Ali Aydınlar University School of Medicine, Istanbul, Türkiye

**Keywords:** robotic surgery, mitral surgery, mitral valve, fibrillating heart, aortic cross-clamping

## Abstract

**Background:**

The primary objective of this study was to evaluate the safety and feasibility of robotic-assisted mitral valve surgery without aortic cross-clamping.

**Methods:**

From January 2010 to September 2022, 28 patients underwent robotic-assisted mitral valve surgery without aortic cross-clamping in our center using DaVinci Robotic Systems. Clinical data during the perioperative period and early outcomes of the patients were recorded.

**Results:**

Most patients were in New York Heart Association (NYHA) class II and III. Mean age and EuroScore II of the patients were 71.5 ± 13.5 and 8.4 ± 3.7 respectively. The patients underwent either mitral valve replacement (*n* = 16, 57.1%) or mitral valve repair (*n* = 12, 42.9%). Concomitant procedures were performed including tricuspid valve repair, tricuspid valve replacement, PFO closure, left atrial appendage ligation, left atrial appendage thrombectomy and cryoablation for atrial fibrillation. Mean CPB times were 140.9 ± 44.6 and mean fibrillatory arrest duration was 76.6 ± 18.4. Mean duration of ICU stay was 32.5 ± 28.8 h and mean duration of hospital stay 9.8 ± 8.3 days. One patient (3.6%) underwent revision due to bleeding. New onset renal failure was observed in one (3.6%) patient and postoperative stroke in one (3.6%) patient. Postoperative early mortality was observed in two (7.1%) patients.

**Conclusions:**

Robotic-assisted mitral valve surgery without cross-clamping is a safe and feasible technique in high-risk patients undergoing redo mitral surgery with severe adhesions as well as in primary mitral valve cases that are complicated with ascending aortic calcification.

## Introduction

Myocardial protection is usually achieved by placing an aortic cross-clamp and delivering cardioplegia in conventional cardiac surgery as well as in robotic-assisted intracardiac repairs. However, this technique represents a clinical challenge in patients undergoing redo cardiac surgery due to adhesions surrounding the heart and the great vessels and also in patients who present with extensive aortic calcification which increases the risk of cerebrovascular accidents and further complications during cross-clamping. The complexity of the clamping and cardioplegia delivery methods has led to the consideration of an alternative approach of hypothermic fibrillatory arrest without the use of the aortic cross-clamp. The primary objective of this study was to evaluate the safety and feasibility of robotic-assisted mitral valve surgery without aortic cross-clamping.

## Materials and methods

From January 2010 to September 2022, 301 patients underwent robotic-assisted mitral valve surgery in our center using Da Vinci Systems (Intuitive Surgical, Inc.) and in 28 of these patients (9.3%), the operation was completed without cross-clamping and on the fibrillating heart. The inclusion criteria to the study were having undergone robotic-assisted mitral valve surgery with or without an additional cardiac procedure and presenting with one or two of the following conditions that increase complication risk for cardiac surgery with aortic cross-clamping, thus being operated under hypothermic fibrillatory arrest: severe aortic calcifications and having had previous cardiac surgery. Concomitant procedures included tricuspid valve repair, tricuspid valve replacement, ablation for atrial fibrillation, left atrial appendage ligation, left atrial thrombectomy and patent foramen ovale (PFO) closure. No patients underwent concomitant coronary artery bypass surgery.

Data for this study which includes medical histories of the patients, demographic characteristics, operative outcomes, electrocardiography analysis and early postoperative outcomes were retrieved from the institutional database retrospectively. Approval from Acibadem Maslak Hospital Institutional Review Board was obtained before establishing the study.

### Surgical technique

Robotic surgery was carried out using the Da Vinci XI or SI systems. Patients with mitral valve disease who required valve replacement (with or without an additional cardiac procedure) due to the disease pathology were included. Myocardial protection was achieved with moderate hypothermia and ventricular fibrillatory arrest. Severe pericardial adhesions due to previous cardiac operations or existence of highly calcified ascending aorta were the indications for fibrillatory arrest during robotic-assisted surgery. Evaluation with routine pre-operative computed tomography was carried out especially in older patients and in patients with a past medical history of atherosclerosis to determine ascending aorta calcification and the need for peripheral cannulation. In addition to the evaluation of aortic calcifications, mitral annular calcification (MAC) was also evaluated during the preoperative computed tomography. If it was found to be extensive, the patient is recommended to undergo a transcatheter mitral procedure. If the calcification was not extensive or if the patient was not eligible for transcatheter options, surgical approach is preferred. In our patient cohort, we had 6 patients with MAC, 4 out of 6 of these patients didn't have extensive calcifications and were operated without any need for resection of the calcifications. In the other 2 patients, the MAC was more extensive but these patients were not eligible for a transcatheter procedure, so neocordae were used for both of them and Alfieri technique was used for one of them.

We assessed the aorta and its peripheral branches for peripheral arterial disease, calcification or thrombotic lesions. The right groin was the site most commonly used if a need for peripheral cannulation was present. We have previously described our surgical setup for peripheral cannulation (1). Ultrasound and transesophageal echocardiograpy guided right common femoral artery and left femoral vein percutaneous cannulation was performed when applicable. For the arterial cannulation, two proglide sutures were used which were positioned perpendicularly to each other before the placement of the arterial cannula. Axillary artery cannulation via surgical exposure was preferred in patients who have atherosclerotic plaques in femoral arteries or at the level of abdominal or thoracic aorta.

**Figure 1:**
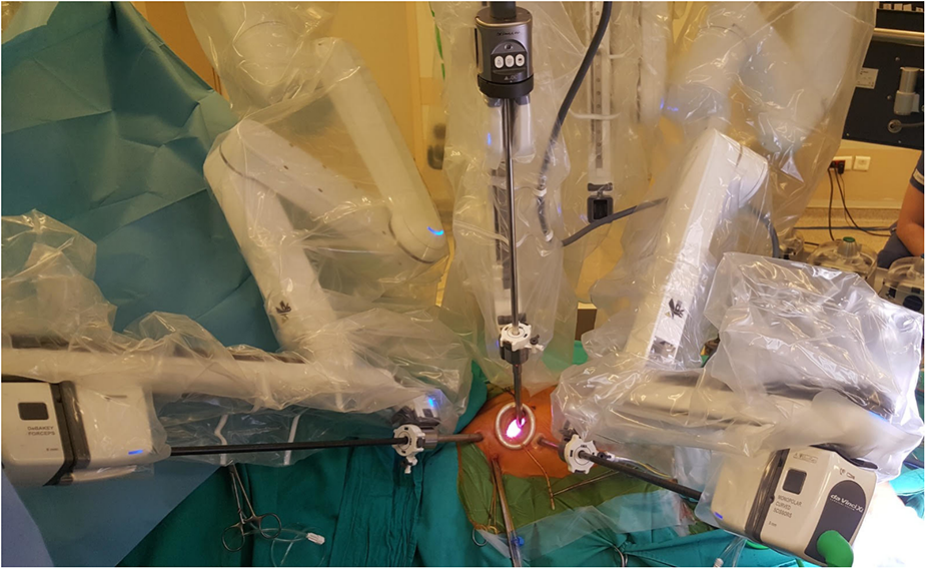
Operative set-up after docking.

The classical set-up for the robotic mitral operations in our center was as described previously (2). A mini thoracotomy is performed after the anesthetic preparation is completed, usually at the right 4th intercostal space and of 4 cm length. Left and right working ports are inserted through the 3rd and 5th intercostal spaces, respectively. A stab incision is made at the 2nd intercostal space through the anterior axillary line and a suction vent and a Chitwood clamp are placed through this incision. The port implantation is followed by the placement of a soft tissue retractor. The robotic system is docked after the trocars are positioned (Figure 1). The camera is sent through the soft tissue retractor and cardiopulmonary bypass (CPB) is started.

After the initiation of CPB the pericardium is opened, an incision of 2 cm length is made anterior and parallel to the phrenic nerve, and further external fixation is performed. Our technique for robotic assisted cardiac surgery without aortic cross clamping was described elsewhere (3). The inferior and superior vena cavae were occluded with bulldog clamps. The fibrillator cable which was delivered through the service port was used to fibrillate the heart. The patients were cooled down to 28°C, and the aim was to keep this level of hypothermia throughout the procedure to prevent any spontaneous conversion to sinus rhythm during surgery. The interatrial groove was dissected, left atriotomy was performed, and an atrial retractor was placed into the left atrium. The LA appendage was ligated in patients with atrial fibrillation (AF) using a double-layered running Prolene 3.0 suture from inside the LA. Following left atriotomy, an extra vent was positioned in the atrium to collect the extra blood and then the exposure was established by robotic retractor.

**Figure 2:**
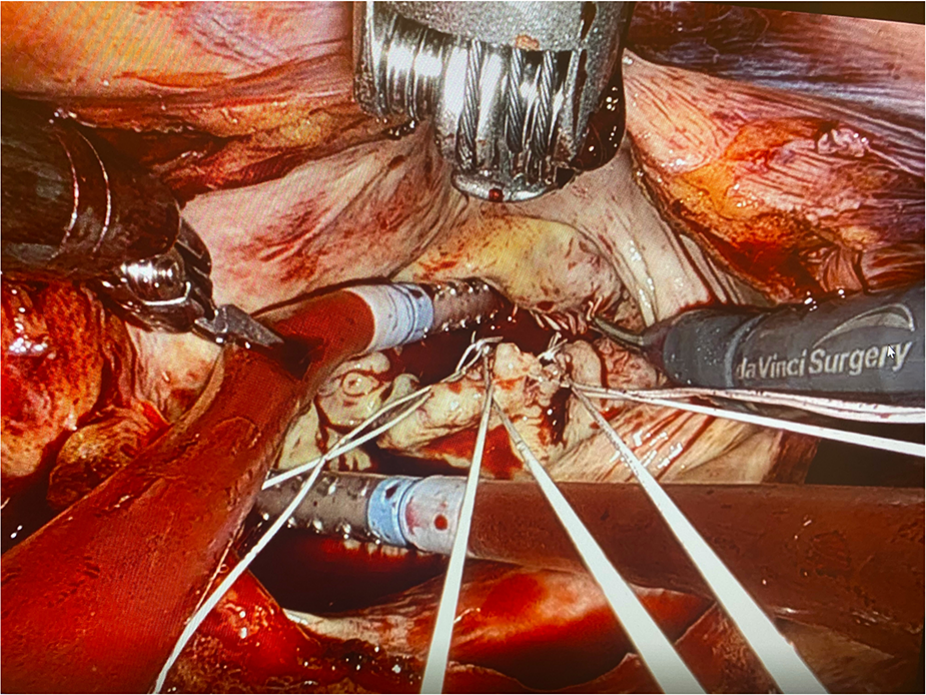
Placing a sump into the left atrium and a sump into the left ventricle.

Carbon dioxide was continuously insufflated (5 L/min) to displace intracardiac air and vacuum assist via venous cannulas was used in case of need. Additionally, the left ventricular catheter helps in avoiding distention of the left ventricle. To ensure adequate exposure during the case, left ventricle was continuously suctioned to prevent distention. This also provides a rather bloodless surgical field. The flow of CPB was increased or decreased at times if needed. All of these techniques help us acquire a better exposure. The rest of the operation was continued in conventional fashion via robotic arms. Deairing is one of the most essential and important parts of the procedure. For this purpose, two suction catheters are used, one of them being kept in the left atrium and the other in the left ventricle via mitral valve (Figure 2). Both catheters are kept in place until the end of CPB and are not removed until no air is visible in transesophageal echocardiogram (TEE). Left atriotomy is closed with two separate prolene sutures from both sides leaving the two suction catheters inside. The sutures are held tight by means of a clamp without knotting. Post Pump TEE is performed to verify proper valve and ventricular function and to ensure complete deairing. Then the suction catheters are removed and the sutures held with clamps are finally knotted after this step.

### Data analysis

All data were presented as mean ± SD, or as percentages. All statistical data analyses were performed using the IBM SPSS Statistics software package 29.0.

## Results

The preoperative demographics of the patients are presented in Table 1. Twenty-eight patients underwent robotic-assisted mitral valve surgery without aortic cross-clamp, 16 (57.1%) men and 12 (42.9%) women with a mean age of 71.5 ± 13.5 years (range 28–92). The patients were in New York Heart Association (NYHA) class I (*n* = 1), II (*n* = 14) and III (*n* = 13). 15 patients (53.6%) had underwent previous cardiac operations.

**Table 1:** Preoperative characteristics of patients.

Demographics	*n* = 28
Age (years)	71.5 ± 13.5
Female gender	12 (42.9)
EuroScore II	8.4 ± 3.7
NYHA Class 2	14 (50)
NYHA Class 3	13 (46.4)
BMI	27 ± 4
Diabetes Mellitus	12 (42.8)
Hypertension	22 (78.6)
Hypercholesterolemia	9 (32.1)
Preoperative AF	10 (35.7)
EF (%)	55.3 ± 9.3
LVEDD (cm)	5.2 ± 0.6
LVESD (cm)	3.5 ± 0.6
Pulmonary artery systolic pressure (mmHg)	56.9 ± 19.2
Creatinine (mg/dl)	1.1 ± 0.4
Previous cardiac procedures	15 (53.6)
**Ethiology of Mitral Valve Pathology**	
Myxomatous/degenerative mitral valve disease	14 (50)
Rheumatic mitral valve disease	6 (21.4)
Infective endocarditis	2 (7.1)
Ischemic mitral valve disease	1 (3.6)
Prosthetic valve dysfunction	5 (17.9)

Data are expressed as mean ± standard deviation or number (%). NYHA, New York Heart Association; BMI, Body Mass Index; AF, atrial fibrillation; EF, ejection fraction; LVEDD, left ventricular end diastolic diameter; LVESD, left ventricular end systolic diameter.

The indications for mitral valve surgery were degeneration (*n* = 14, 50%), rheumatic valve (*n* = 6, 21.4%), infective endocarditis (*n* = 2, 7.1%), ischemia (*n* = 1, 3.6%) and prosthetic valve dysfunction (*n* = 5, 17.9%). The patients underwent either mitral valve replacement (*n* = 16, 57.1%) or mitral valve repair (*n* = 12, 42.9%). Amongst 16 patients who underwent mitral valve replacement, 8 (50%) received a mechanical valve and 8 (50%) a bioprosthetic valve.

Concomitant procedures were performed including tricuspid valve repair, tricuspid valve replacement, PFO closure, left atrial appendage ligation, left atrial appendage thrombectomy and cryoablation for atrial fibrillation. Concomitant tricuspid valve surgery was performed in 11 (39.3%) of the patients. Tricuspid repair was performed in ten (35.7%) and tricuspid valve replacement was performed in one (3.6%) patient within this group. There were ten patients with preoperative atrial fibrillation and ablation surgery for atrial fibrillation was performed for six of these patients. Operative data is summarized in Table 2. Mean CPB times were 140.9 ± 44.6 and mean fibrillatory arrest duration was 76.6 ± 18.4. The mean mechanical ventilation time was 8.3 ± 5.3 h, mean duration of ICU stay was 32.5 ± 28.8 h and mean duration of hospital stay 9.8 ± 8.3 days. Mean drainage in ICU was 334.6 ± 270.2 ml, and one patient (3.6%) underwent revision due to bleeding. New onset renal failure was observed in one (3.6%) patient in the postoperative period. Autotransfusion was used in all patients. There was no case of conversion to open thoracotomy or sternotomy. Transient or permanent AV block was not observed in any of the patients.

**Table 2:** Operative data.

Variables	Patients (*n* = 28)
**Mitral Surgery**	
Mitral valve repair	12 (42.9)
Mitral valve replacement	16 (57.1)
**Mitral prosthesis**	
Mechanical valve	8 (50)
Biological valve	8 (50)
**Concomitant Procedures:**	
Tricuspid valve replacement	1 (3.6)
Tricuspid valve repair	10 (35.7)
PFO Closure	2 (7.1)
LAA Ligation	7 (25)
Cryoablation	6 (21.4)
LAA Thrombectomy	1 (3.6)
**Operative duration (min):**	
Fibrillatory arrest	76.6 ± 18.4
Cardiopulmonary bypass	140.9 ± 44.6

Data are expressed as mean ± standard deviation or number (%). PFO, patent foramen ovale; LAA, left atrial appendage.

Postoperative early mortality was observed in two (7.1%) patients, the first due to a pulmonary infection and the second due to a cerebrovascular accident alongside GI ischemia causing multiorgan failure. The cerebrovascular event occurred due to hemodynamic instability accompanying multiorgan failure. Postoperative clinical outcome data is presented in Table 3.

**Table 3:** Postoperative outcomes.

Outcomes	*n* = 28
Mechanical ventilation time (h)	8.3 ± 5.3
ICU Stay (h)	32.5 ± 28.8
**Inotrope Support**	
None	22 (78.6)
Low Dose	1 (3.6)
High Dose	5 (17.9)
Blood transfusion requirement	3 (10.8)
Postoperative atrial fibrillation	6 (21.4)
Transient AV Block	0 (0)
Permanent AV Block	0 (0)
Postoperative stroke	1 (3.6)
Postoperative dialysis	1 (3.6)
**Infection**	
Pneumonia	1 (3.6)
Urosepsis	1 (3.6)
Chest tube drainage in ICU (ml)	334.6 ± 270.2
Reoperation	1 (3.6)
Hospital stay (days)	9.8 ± 8.3
Reintubation	4 (14.3)
30-day mortality	2 (7.1)

Data are expressed as mean ± standard deviation or number (%). ICU, intensive care unit.

## Discussion

Our study involved 28 patients undergoing robotic-assisted mitral valve surgery with or without concomitant cardiac procedures, moderate hypothermic CPB, and no aortic clamping. The technique for mitral valve surgery without aortic cross-clamping has been reported previously (4–9) with good results as an alternative to conventional redo-sternotomy and also in patients with severe aortic calcification. In a study by Holman et al. which included 84 patients undergoing reoperative mitral valve surgery that compares myocardial management methods, the mortality of patients who received cardioplegic arrest with aortic cross clamping was significantly higher than patients who had mitral surgery with ventricular fibrillation or beating heart technique (6).

In other studies reporting the use of cross clamp in reoperative cases for mitral surgery, the prevalence of neurological adverse events were reported to be up to 7% (10, 11). A study by Maselli and colleagues evaluating micro embolic event occurrence during minimally invasive mitral valve procedures showed that aortic manipulation with cross-clamping is associated with a significant increase in brain embolic event rate (12). Postoperative stroke was observed in only one patient (3.6%) in our study. Avoidance of cross-clamping and complete de-airing to prevent air emboli are factors that are thought to be contributing to the low rate of neurological adverse events in this study.

Robotic cardiac surgery follows an upward curve worldwide as a greater variety of cardiac procedures are done with good clinical outcomes in a minimally invasive manner with this technology. In the paper by Cerny et al. which presents a multicenter analysis of robotically assisted cardiac surgery in Europe, it is shown that the conversion rates are convincingly low and that clinical outcomes are favourable (13). Better postoperative outcomes have also been demonstrated with robotic mitral valve surgery compared to conventional mitral valve replacement before (14–16). The combination of robotic surgery with electrically induced ventricular fibrillation was used for both primary and redo mitral valve cases in this study. We demonstrated that a robotic-assisted approach without aortic cross-clamping may provide reasonable perioperative outcomes in this specific group of patients undergoing mitral valve surgery. Our mortality rate of 7.1% was in accordance with the the average STS-predicted operative mortality for the present patient cohort when using a conventional approach, which is around 7% (7). Overall, our results demonstrate a low incidence of perioperative complications and a fast recovery.

Excellent exposure of the mitral valve is achieved via right-sided robotic assisted surgery and the use of ventricular fibrillation eliminates the need for extensive mediastinal dissection required to place the aortic cross clamp during redo operations. Potential complications of aortic cross-clamping such as cerebral thromboemboli and those of repeat sternotomy such as catastrophic reentry into the heart which may cause massive bleeding or injury to previous bypass grafts may be avoided by this approach. In patients undergoing redo operations, robotic surgery also presents an advantage of better handling of the adhesions from previous operations, better visualization of the mitral valve and a smaller thoracotomy for better cosmetic results.

Two crucial points that need consideration with this technique is to ensure complete de-airing to prevent air embolisms and to prevent left ventricular distention which may result in subendocardial ischemia. Higher risk of stroke has been reported in a study by Svensson et al. in patients undergoing mitral valve operations on a fibrillating heart through a right thoracotomy compared to those undergoing surgery via a redo-sternotomy approach (17). Just as in minimally invasive surgery, robotic surgery can be complicated by air embolisms due to the difficulty in performing standard de-airing maneuvers. To avoid this complication, continuous CO_2_ insufflation (5 L/min) was used through the operation. Heart was kept fibrillating while it is open to air to prevent air ejection. An extra venting sucker was placed across the mitral valve to prevent left ventricular distention and was left in place during rewarming to facilitate de-airing. Axillary cannulation instead of femoral was carried out in patients with peripheral atherosclerotic disease to avoid embolic stroke. After weaning from CPB, complete de-airing was ensured by TEE.

Our study presents potential limitations. First of all, the study is retrospective, nonrandomized by nature and contains a limited number of patients. This leads to a difficulty in obtaining direct conclusions from this study. An extended patient follow-up will aso be needed to evaluate the long term outcomes of the above mentioned surgical technique.

## Conclusion

Robotic-assisted mitral valve surgery without cross-clamping and under fibrillation represents a good alternative to conventional mitral valve surgery with cross-clamping and cardioplegic arrest. Our results suggest that the technique is safe and feasible in high-risk patients undergoing redo mitral surgery with adhesions as well as in primary mitral valve cases that are complicated with ascending aortic calcification.

## Data Availability

The raw data supporting the conclusions of this article will be made available by the authors, without undue reservation.
